# Out-of-pocket payment and catastrophic health expenditure of tuberculosis patients in accessing care at public-private mix clinics in Myanmar, 2022

**DOI:** 10.1186/s40249-024-01248-7

**Published:** 2024-11-05

**Authors:** Myat Noe Thiri Khaing, Nandi U, Luu Maw, Htet Arkar, Saw Pa Pa Naing, May Me Thet

**Affiliations:** 1 Population Services International Myanmar, Yangon, Myanmar; 2 Sun Community Health Myanmar, Yangon, Myanmar

**Keywords:** Tuberculosis, Patient cost, Catastrophic cost, Private health sector, Care seeking, Myanmar

## Abstract

**Background:**

The financial burden of tuberculosis (TB) can hinder patients and their families, creating obstacles throughout the care cascade, despite TB prevention and control being provided free of charge. In Myanmar, patients can visit private providers operating under public-private mix (PPM) schemes, where TB services (diagnosis and treatment) are typically offered at no cost. The study focused on quantifying the financial burden faced by TB patients seeking care from Myanmar's PPM providers.

**Methods:**

This cross-sectional telephone survey included 695 adults seeking TB treatment [drug-susceptible TB (DS-TB) and retreatment TB] from various private providers in four states and regions with high TB burden in Myanmar. Telephone interviews were conducted in May and June 2022. Both direct and indirect costs incurred from the patient and their household perspective were valued in 2022 and estimated throughout pre- and post-TB treatment episodes. The TB-affected households were defined as experiencing catastrophic health expenditure if their expenditure due to TB exceeded 20% of their capacity to pay, as recommended by the World Health Organization. All cost data were collected in Myanmar Kyats (MMK) and converted to USD (1 USD = 1850 MMK as of July 20, 2022). Logistic regression analysis was done to identify the determinants of catastrophic health expenditure.

**Results:**

The findings showed patients made a median of 7 times for clinic visits throughout their treatment, with the median total cost for the entire TB treatment being 53.4 US dollars (USD), including direct medical and testing costs (11.9 USD) and direct non-medical patient expenditure (11.6 USD). Pre-treatment costs were higher compared to post-treatment costs (the intensive phase and continuation phase). During the intensive phase, TB care cost was nearly free, but during the continuation phase, it was a median of 2.6 USD. About 34.5% of patients experienced catastrophic health expenditure due to TB treatment, with expenses exceeding 20% of their capacity to pay. Multivariate regression analysis revealed that patients with a history of hospitalization (a*OR* = 14.84; *P* < 0.01), seeking care from regions other than Yangon (a*OR* = 2.6; *P* < 0.01), and using coping strategies (a*OR* = 12.53; *P* < 0.01), were more likely to face catastrophic financial burdens. Higher monthly household income (over 162 USD) was associated with a decreased risk of incurring catastrophic health expenditure (a*OR* = 0.38; *P* < 0.01).

**Conclusions:**

TB patients and their households in Myanmar faced risk of catastrophic costs, even when treated in the private sector with free diagnostic charges and anti-TB medicine. The study highlighted the need for additional strategies or policies to make TB care affordable and mitigate the financial burden of TB-affected households.

**Graphical Abstract:**

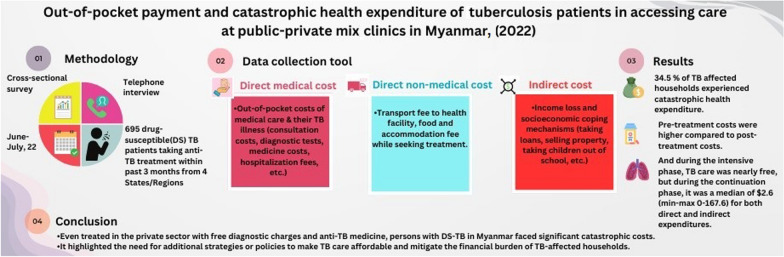

## Background

Tuberculosis (TB) remains among the major global public health problems [1]. Out of many countries suffering from TB, Myanmar is listed as one of 30 countries facing a high burden of TB and related conditions [2]. According to the World Health Organization (WHO), TB incidence in Myanmar was 360 in 100,000 population in 2021 [3, 4].

In addition to TB being one of the deadliest infectious diseases, it also imposes not only a physical but also a significant financial burden on affected individuals and their households [5]. The financial burden is particularly severe for multi-drug-resistant TB (MDR-TB) cases [6]. Income loss due to illness, high cost of treatment, disruption of daily life, and many adverse effects are some of the factors that have made it increasingly difficult to treat such cases [7, 8].

Public–private partnerships (PPM) have been recognized as a potential solution to alleviate this burden by improving access to affordable TB diagnosis and treatment [9]. Private general practitioners (GPs) affiliated with non-profit organizations play a crucial role in Myanmar's National TB Program, contributing significantly to case finding and care quality. In 2019, PPM initiatives contributed 32% of all TB notifications in Myanmar [10]. The Ministry of Health and Sports in Myanmar developed guidelines in 2003 to involve private general practitioners in the National TB Program, which helped to increase case finding and quality of care. A total of 2965 GPs were involved in TB activities at the end of 2018, and the PPM accounted for 25% of total case notifications in 2020 [11].

Prior research in Myanmar has mainly focused on the quality of care and treatment outcomes, with limited study population and geographic coverage [12]. To date there have been no studies on catastrophic cost done in private sector and there is a limited understanding of the financial aspects of TB treatment in the private sector, as the previous studies have primarily focused on public facilities [13].

This study aimed to fill this gap by exploring the catastrophic health expenditure (CHE) among TB-affected households in private sector clinics. The study provided valuable baseline information for policymakers and decision-makers in Myanmar, aiding in the enhancement of the private healthcare financing system and PPM interventions.

## Methods

### Study design and setting

A cross-sectional quantitative study was done with drug-susceptible TB (DS-TB) patients and retreatment-TB patients currently undergoing treatment at 163 private clinics in Kachin, Kayin, Yangon and Ayeyarwady region, of Myanmar, selected for their high TB caseloads and concentration of private healthcare providers in these areas [14]. This study was part of a larger assessment which was to conduct the private sector providers’ landscaping and their role in TB care in high burden areas. In the project, the selection of areas was based on existing tuberculosis project implementation in private sector and the selected areas had no or little TB private sector providers.

The private sector providers in Myanmar are essential partners in TB care, with the potential to enhance access to services, improve patient outcomes, and contribute to broader public health efforts against tuberculosis. While the care and management of drug-resistant TB (DR-TB) are mainly operated by the national TB control programme and public health sector, our study specifically focused on drug-susceptible TB (DS-TB) patients seeking care in the private sector.

Our study participants were drug-susceptible TB patients receiving treatment from private GPs, who had partnerships with non-governmental organizations. Specifically, the private GPs collaborated with TB PPM partners like Population Services International (PSI), as well as other TB implementing partners such as Medical Action Myanmar (MAM), the Asian Harm Reduction Network (AHRN), and the Myanmar Anti-TB Association (MATA) which played crucial roles in the TB healthcare landscape within the study regions.

The study utilized a standardized questionnaire from the World Health Organization (WHO) to collect data [15]. This questionnaire gathered information on cost components such as direct medical, direct non-medical, and indirect costs incurred by TB patients. It also aimed to capture details about the coping mechanisms employed by patients.

### Study population and eligibility

Participants eligible for the study were 18 years or older, diagnosed with drug-susceptible tuberculosis, and receiving care (Initial or retreatment) from public-private mix (PPM) clinics. Recruitment occurred between December 2021 and April 2022. Both patients and healthcare providers received information about the study and obtained initial consent from potential participants. The research team obtained a weekly list of patients, contacting them to arrange for telephone interviews.

As of January 1st, to April 1st 2022 report, there were 1860 eligible participants for the study from 163 private sector clinics within the four states and regions. 62.6% (1165 TB patients) were being excluded due to completed treatment, age under 18, serious illness, death or loss to follow-up, and incomplete data. Various contact issues such as phone power-off, no answer, wrong number, refusals, and completed TB treatment were the primary reasons for exclusion.

### Sample size

Study samples were selected from TB patient lists provided by five TB implementing partners receiving anti-TB treatment at private clinics from January 1st to April 1st, 2022. The sample size (*n* = 700) was estimated considering a 15% nonresponse rate and private sector's presumed contribution of 18.9% to total TB cases, as reported in the 2018 National TB program report. A systematic random sampling approach with a design effect of 1.5 was applied by using the following formula [16].

$$ {\text{Sample size}} = \frac{{\frac{{z^{2} \times p\left( {1 - p} \right)}}{{e^{2} }}}}{{1 + \left( {\frac{{z^{2} \times p\left( {1 - p} \right)}}{{e^{2} N}}} \right)}} $$

where *e* = margin of error (0.03), and *z* = z-score (1.96), *p* = proportion of total TB cases contribution by private sector (0.188), and *N* = population size (136,039) based on the 2018 NTP report [17].

The number of samples required from each state and each IP was determined based on probability proportional to the size (PPS) of the total patient list collected in a defined period. Among the listed patients, 1860 selected patients were approached for telephone interviews. In the end, 695 TB patients (37.4%) were successfully interviewed, while the remaining 1165 TB patients (62.6%) were unable to be reached due to registered phone numbers being logged off, not answered, wrong number, refused, patients passing away, treatment being completed, and interrupted interview. The participation rate was 99.3% anticipated by the survey design (695/700).

### Definitions

Household socio-economic status information such as household assets, housing materials, drinking water source, etc. was collected through a short version of equity tool [18].

Cost analysis, from the patients' perspective, covered various financial aspects. Direct medical costs included payments for consultations, tests, medicines, and medical procedures, while direct non-medical costs included expenses like transportation and accommodation. Costs were calculated separately for different stages of treatment: pre-TB treatment, and post-TB treatment (intensive treatment, and continuation treatment) [15].

Hospitalization costs included all expenditures related to a patient's hospital stay for patients and accompanying family or friends [19]. Indirect costs included productivity loss (the loss of personal income due to TB illness) [19], and coping costs (selling assets and borrowing money).

Indirect costs were assessed using a human capital approach. This method was chosen due to the unreliable household income data reported by patients and the high proportion of patients engaged in informal employment compared to other sectors in Myanmar. Indirect costs were determined by multiplying the reported hours spent seeking and receiving care during the TB episode by the individual’s hourly income. This approach accounted for time lost traveling to health facilities and waiting during healthcare consultations for both patients and their caregivers. The total self-reported time spent on these activities was multiplied by the estimated hourly income per person. Hourly income was estimated from the self-reported income data collected from all survey participants, calculated based on their reported individual income and hours worked.

The food-share-based poverty line was set using the proportion of household total expenditure that a household allocated to food [24]. A household was classified as poor if its total expenditure was less than its calculated subsistence spending. Conversely, a household was considered non-poor if its total expenditure was equal to or greater than its subsistence spending. The poverty line (PL) in this calculation was defined as the subsistence expenditure per equivalent capita, which was calculated by (1) identifying the food expenditure share of households, specifically those in the 45th to 55th percentile range of food expenditure as a share of total household expenditure; (2) calculating the weighted average of food expenditures within this range; and (3) determining the minimum standard of income necessary to meet the basic needs of a household of equivalent size.

Catastrophic heath expenditure occurred when a household’s total out-of-pocket health payments equal or exceed 20% of household’s capacity to pay or non-subsistence spending [20, 21]. This threshold was used to identify households facing financial burden due to TB-related expenses.

### Data collection

Data collection took place in May and June 2022 using a WHO-adapted questionnaire translated into Myanmar [15, 21] and was created in CSPro® (Census Bureau, USA). The telephone survey covered TB patient information on demographic details, economic status, current TB treatment, direct medical and non-medical payments, indirect costs (income loss or time), caregiver costs, coping mechanisms, household assets and income during post-TB treatment phase (e.g., either the intensive phase or the continuation phase). The questionnaire was pretested with TB patients and the PSI/Myanmar staff to test fluency of the questions and then they were modified accordingly. All the duration for completing one interview lasted 45–60 min.

Interviews were done via telephone calls. The study collected data and checked for completeness and consistency using the Stata version 14.2 (StataCorp, College Station, TX, USA). Access to the secure server was limited to field management staff. Moreover, these uploaded electronic data were stored in password-protected devices. The PSI Myanmar research team had access to this information for analysis and research purposes.

### Participant recruitment

For the recruitment, PSI Myanmar initially informed private health care providers and TB implementing partners via phone or email, usually 1–2 weeks in advance, seeking their approval to recruit their clients. The TB patient information was collected by using the client listing form, including mostly data elements from the clinic register or provider records, such as patient ID or name, patient phone number, age, type of TB, residential township, and current treatment status.

### Consent for interview

Before participants were recruited for the study, all participants were asked for their verbal consent for the interview. The interview session happened after receiving their consent. The contact information for those not consenting was deleted immediately.

### Data analysis

The data were exported to Stata version 14.2 (StataCorp, College Station, TX, USA) for cleaning and analysis. The socio-economic status of households, ranging from the poorest to the richest, was determined using a set of questions about household assets [18]. This tool measured the relative wealth of a household by converting its assets into a composite score and applying a cut-off to establish five wealth quintiles [22]. The cut-offs applied were those from national quintiles, therefore, the wealth of studied households represented their relative wealth with reference to national wealth quintiles [23].

All cost data were collected in Myanmar Kyats and converted to USD (1 USD = 1850 Myanmar Kyats as of July 20, 2022). Descriptive statistics, including median, mean, inter quartile range (IQR), and 95% confidence interval (*CI*) for continuous data, as well as frequencies for categorical data, were used. Continuous variables with normal distribution were presents as mean ± standard deviation (SD); non-normal variables were reported as median (IQR). Costs were analyzed from the patient's perspective, including direct and indirect costs. For each treatment stage, costs per visit were calculated, and total costs were estimated using median costs with ranges. Median values were used due to data skewness.

The patient costs were separately calculated for the stages of treatment pre-TB treatment, and post-TB treatment (intensive treatment, and continuation treatment). For patients with two to six months of treatment, total costs for the continuation phase were computed by summing actual visit costs and estimated costs calculated by multiplying their average treatment cost per visit based on remaining visits. The latter estimate was based on their remaining treatment duration and clinic visit frequency to get the best estimate. For patients in the intensive phase (< 2 months), average continuation phase costs (estimated cost between two and six months) were added to their initial two months for the entire six-month duration.

Statistical significance was defined as *P* < 0.05. The out-of-pocket payments were calculated as sum of direct and indirect TB treatment expenditures, including productivity and coping costs due to TB. Catastrophic health expenditure (CHE) was defined as total health expenditure equaling or exceeding 20% of the household's capacity to pay [3, 15, 20, 21, 24]. Moreover, the variable threshold levels were considered: 40%, 30% and 10%. Additionally, we explored different outcome measures, such as the human capital and output approaches, to provide a more comprehensive analysis.

Bivariate and multivariable logistic regression analysis were used to identify variables significantly associated with CHE. The variables included patients' age, gender, regions, total monthly household income, national SES quintile, treatment category, history of hospitalization, history of coping strategy use, health-seeking channel, and comorbidities. *P*-values less than 0.05 and adjusted odd ratios (a*OR*) with 95% *CI* were reported.

## Results

### Participant characteristics and health-seeking

Table 1 highlights the sociodemographic characteristics of study participants. The majority 457 (65.8%) lived in urban areas. Among them, 26.2% were 18 to 30 years (43.6 ± 15.9 years), 58.4% were male, 53.8% were middle-high school level, 46.6% were employed, 53.1% earned less than 300,000 MMK or 162 USD monthly. 441 (63.5%) were married, and 45.3% of households fell in the poorest wealth quintile in reference to national wealth quintile. Regarding smoking history in past 12 months, 56.3% were non-smokers, 31.9% were ex-smokers, and 11.8% were current smokers.

**Table 1:** Socio-demographic and clinical profile of survey participants

	Urban (*n* = 457)		Rural (*n* = 238)		Total	
	*n*	%	*n*	%	*N*	%
Age group of the study participants						
< 30 years	131	28.7	51	21.4	182	26.2
31–40 years	103	22.5	41	17.2	144	20.7
41–50 years	93	20.4	40	16.8	133	19.1
51–60 years	65	14.2	45	18.9	110	15.8
Over 60 years	65	14.2	61	25.6	126	18.1
Age, mean (*SD*), years	41.8 (15.4)		47 (16.5)		43.6 (15.9)	
Gender						
Male	262	57.3	144	60.5	406	58.4
Female	195	42.7	94	39.5	289	41.6
Education level						
Lower than primary school	112	24.5	123	51.7	235	34
Middle and high school	267	58.4	108	45.4	375	53.8
Higher than high school	78	17.1	7	2.9	85	12.2
Employment status						
Employed with earnings	232	50.8	92	38.7	324	46.6
Unemployed or no job	146	31.9	98	41.2	244	35.1
Dependent and unable to work	79	17.3	48	20.2	127	18.3
Marital status						
Single	124	27.1	47	19.7	171	24.6
Married	279	61.1	162	68.1	441	63.5
Divorced/Separated	20	4.4	6	2.5	26	3.7
Windowed	33	7.2	23	9.7	56	8.1
Refused to answer	1	0.2	0	0	1	0.1
State/Region						
Ayeyarwady	11	2.4	49	20.6	60	8.6
Kachin	16	3.5	12	5	28	4
Kayin	47	10.3	*39*	16.4	86	12.4
Yangon	383	83.8	138	58	521	75
Total monthly household (HH) income						
≤ 300,000 MMKs or 162 USD	223	48.8	146	61.3	369	53.1
> 300,000 MMKs or 162 USD	234	51.2	92	38.7	326	46.9
Household Wealth Quintiles (Ref: National Quintile)						
Poorest	10	2.2	26	10.9	36	5.2
Second poorest	25	5.5	44	18.5	69	9.9
Middle	42	9.2	42	17.6	84	12.1
Second richest	120	26.3	71	29.8	191	27.5
Richest	260	56.9	55	23.1	315	45.3
Smoking status (within past 12 months)						
Smoker	52	11.4	30	12.6	82	11.8
Non-smoker	263	57.5	128	53.8	391	56.3
Ex-smoker	142	31.1	80	33.6	222	31.9
Type of TB						
Pulmonary TB	429	93.9	230	96.6	659	94.8
Extra pulmonary TB	28	6.1	8	3.4	36	5.2
TB patients’ responses to treat the symptoms at first						
Modern medicine (pills, drug cocktail, etc.)	162	35.4	109	45.8	271	39.0
Go to clinic	195	42.7	62	26.1	257	37.0
Do nothing	62	13.6	38	16	100	14.4
Traditional medicine	37	8.1	19	8	56	8.1
Homemade remedies (Herbs/tea, etc.)	1	0.2	10	4.2	11	1.6
The place where they first diagnosed as confirmed TB case						
Private GP clinics and INGO clinics	377	82.5	191	80.3	568	81.7
Private hospital	38	8.3	13	5.5	51	7.3
Public hospital, health center	38	8.3	27	11.3	65	9.4
Community providers, health assistants, nurses, Midwives	2	0.4	5	2.1	7	1
Others: Informal providers, Pharmacy, relative, friend	2	0.4	2	0.8	4	0.6
Duration of first diagnosed as having TB (in years)						
< 5 years	362	79.2	213	89.5	575	82.7
5–10 years	7	1.5	4	1.7	11	1.6
> 10 years	31	6.8	11	4.6	42	6
Do not remember	57	12.5	10	4.2	67	9.6
Treatment regimen						
Initial regimen	390	85.3	210	88.2	600	86.3
Retreatment regimen	67	14.7	28	11.8	95	13.7
Duration of taking the anti-TB medicine (in months)						
At least 2 months	8	1.8	0	0	8	1.2
2–6 months	207	45.3	127	53.4	334	48.1
More than 6 months	191	41.8	102	42.9	293	42.2
Don’t know	51	11.2	9	3.8	60	8.6
HIV status						
Positive	22	4.8	10	4.2	32	4.6
Negative	364	79.6	193	81.1	557	80.1
Don’t know	71	15.5	35	14.7	106	15.3
Having other comorbidities [such as diabetes mellitus (DM), hypertension (HTN)]						
Yes	86	18.8	45	18.9	131	18.8
No	314	68.7	168	70.6	482	69.4
Don’t know	57	12.5	25	82	82	11.8

*TB* Tuberculosis, *GP* General practitioner, *INGO* International non-governmental organization

Among interviewed patients, 94.8% had pulmonary smear-positive TB. To alleviate symptoms, 39% self-administered medicine, while 37% went to the clinic before seeing healthcare providers. Diagnosis occurred mostly at private clinics (81.7%), followed by public hospitals and health centers (9.3%). The majority (82.7%) were diagnosed less than 5 years ago, and 95(13.7%) were on retreatment. About 48.1% took anti-TB medicine for 2–6 months, and 1.2% for at least 2 months. Among the patients interviewed, 32 (4.6%) were HIV positive, and 131 (18.8%) had comorbidities such as diabetes mellitus and hypertension (Table 1).

### TB affected household cost for TB diagnosis and treatment (pre-TB treatment and post-TB treatment)

The median number of healthcare visits for the entire treatment was 7.0 (Min–Max: 1–30). The median cost for the entire treatment was 98,700 MMK (53.4 USD). The patient cost included medians of 22,000 MMK (11.9 USD) for direct medical costs and 21,500 MMK (11.6 USD) for direct non-medical patient expenditure (Table 2).

**Table 2:** Breakdown of cost analysis for TB treatment phases and categories

Clinic visits and cost per patient by cost type and TB treatment phase	Total grand cost for the whole treatment duration (*N* = 695)	Total cost before TB treatment (*n* = 665)	Total cost for the intensive treatment duration (*N* = 695)	Total cost for the continuous treatment duration (n = 310)
	Median (Min–Max)	Median (Min–Max)	Median (Min–Max)	Median (Min–Max)
Number of clinic visits	7 (1–30)	3 (1–24)	2 (1–9)	2 (1–9)
Grand total cost for the whole treatment^a^	98,700 (0–16,004,500) MMK 53.4 (0–8651.1) USD	31,000 (0–1,382,500) MMK 16.8(0–747.3) USD	0 (0–140,000) MMK 0 (0–75.7) USD	5500(0–310,000) MMK 2.6(0–167.6) USD
Direct medical cost	22,000 (0–3,532,000) MMK^b^ 11.9 (0–1909.2) USD	16,000 (0–1,310,500) MMK 9 (0–708) USD	0 (0–598,000) MMK 0 (0–323) USD	0 (0–50,000) MMK 0(0–27) USD
Direct non-medical cost	21,500 (0–1,810,000) MMK^b^ 11.6 (0–978.4) USD	5000 (0–468,000) MMK 3(0–253) USD	5000 (0–190,000) MMK 3 (0–103) USD	6000 (0–140,000) MMK 3.4 (0–76) USD
All caregiver cost	0 (0–139,000) MMK 0 (0–75.1) USD	0 (0–81,000) MMK 0 (0–44) USD	0 (0–77,000) MMK 0 (0–42) USD	0 (0–50,000) MMK 0 (0–27) USD
Productivity loss for both patient and caregiver	0 (0–420,000) MMK 0 (0–227.0) USD	0 (0–230,000) MMK 0 (0–124) USD	0 (0–140,000) MMK 0 (0–76) USD	0 (0–240,000) MMK 0 (0–130) USD
Coping cost^c^	0 (0–16,000,000) MMK 0 (0–8649) USD	NA	NA	NA

^a^Grand total costs: Total direct costs + total indirect costs, 1 USD = 1850 MMK

^b^Including cost during hospitalization

^c^Coping cost was asked for the whole phase of TB treatment

Before TB treatment, the median direct medical cost was 16,000 MMK (9 USD) (Min–Max: 0–1,310,500 MMK, 0–708 USD). During the intensive (2 months) and continuation (6 months) treatment phases, direct medical costs were almost free. The median direct non-medical cost before treatment was 5000 MMK (3 USD), ranging from 0 to 468,000 MMK (0–253 USD). During intensive and continuation phases, the median direct non-medical cost was 5000 MMK (3 USD) and 6000 MMK (3.4 USD). Zero caregiver costs and productivity losses were reported throughout all treatment phases (before TB treatment, post TB treatment (intensive and continuation treatment) (Table 2).

Table 3 highlighted the disparities in TB-related healthcare costs across different household income quintiles. Higher-income quintiles had higher median household expenditures and a higher capacity to pay for healthcare expenses. The median cost for their capacity to pay was 178,618 MMK (96.6 USD), and the out-of-pocket health payments share of household capacity to pay (oopctp) was 11.1%. Across the different quintiles, it showed that the poorest quintile had the highest oopctp at 36.7%, and the poorest quintile had the highest percentage of poor households at 27.8%. About 34.5% of TB affected households experienced catastrophic health expenditure. This indicated that even some non-poor households in higher SES quintiles were affected by TB related poverty, and lower-income households faced a higher cost burden for TB care compared to higher-income households.

**Table 3:** Total monthly expenditure and health expenditure indicators during tuberculosis (TB) treatment (*N* = 695)

	Indicators	Catastrophic cost Including direct, indirect, productivity and coping costs	Poorest quintile (*n* = 36)	Second poorest quintile (*n* = 69)	Middle quintile (*n* = 84)	Second richest quintile (*n* = 191)	Richest quintile (*n* = 315)
3.1	Total monthly household expenditures [Median (Min–Max)]	318,250 (3167–3,267,417) MMK 172.0 (2–1766) USD	212,875 (66,500–526,000) MMK 11.8 (35.9–284.3) USD	249,833 (3167–923,167) MMK 135.0 (1.7–499.0) USD	285,917 (5167–927,533) MMK 154.5 (2.8–501.1) USD	294,417 (11,833–1,746,333) MMK 159.1 (6.4–94.0) USD	371,500 (95,100–3,267,417) MMK 200.8 (51.4–1766.2) USD
3.2	Household 's capacity to pay (ctp) [Median (Min–Max)]	178,618 (3167–3,140,289) MMK 96.6 (1.7–1697.5) USD	88,333 (19,000–338,579) MMK 47.7 (10.3–183.0) USD	140,899 (3167–833,167) MMK 76.2 (1.7–450.4) USD	149,619 (51,667–778,183) MMK 80.9 (28–420.6) USD	150,000 (11,833–1,596,983) MMK 81.1 (6.4–863.2) USD	229,870 (22,500–3,140,289) MMK 124.3 (12.2–1697.5) USD
3.3	Out-of-pocket health payments share of household capacity to pay (oopctp) (%)	11.1	36.7	22.2	14.3	10.9	7.4
3.4	Percentage of poor household	8.1	27.8	15.9	10.7	7.3	3.8
3.5	Percentage of household with catastrophic health expenditure (cata)	34.5	77.8	53.6	39.3	36.1	23.2
3.6	Percentage of non-poor household impoverished by health payments	3.6	23.1	5.2	4.0	5.1	0.7

1 USD = 1850 MMK

Table 4 showed the prevalence of CHE across various threshold levels using both the human capital and output approaches. The findings suggested that the human capital approach was more sensitive at capturing the financial strain at lower threshold levels, while the output approach became relatively more significant at higher thresholds. It was noted that the human capital approach generally identified a higher percentage of households facing catastrophic health expenditure, particularly at the 10% and 20% thresholds. However, as the threshold levels increased to 30% and 40%, the differences between the two approaches decreased. At the 30% threshold, both methods recognized nearly the same proportion of households experiencing CHE. Interestingly, at the 40% threshold, the output approach exceeded the human capital approach by identifying a greater number of households experiencing catastrophic expenditure (Table 4).

**Table 4:** Prevalence of catastrophic health expenditure at variable thresholds by different approaches

Catastrophic health expenditure	Threshold level, standard WHO approach				Total
	10% (*N*, %)	20% (*N*, %)	30% (*N*, %)	40% (*N*, %)	
% Using the output approach	(279, 40.14)	(204, 29.35)	(172, 24.75)	(142, 20.43)	695
% Using the human capital approach	(368, 52.95)	(240, 34.53)	(174, 25.04)	(112, 16.12)	

### Factor associated with catastrophic cost among TB patients

In binary logistic regression, many variables were statistically significant to catastrophic cost: household income over 300,000 MMKs (162 USD) (*P* < 0.01), unemployment (*P* = 0.01), hospitalization (*P* < 0.01), using coping strategies (*P* < 0.01), and seeking care from non-Yangon areas (*P* < 0.01). Multivariate logistic regression analysis found that TB-affected households with a history of hospitalization [adjusted odds ratio (*OR*) = 14.84;* P* < 0.01] compared to the reference those without such history, using coping strategies such as selling properties, jewelry, borrowing money (a*OR* = 12.53; *P* < 0.01) compared to those that did not do so, and those from non-Yangon areas (a*OR* = 2.6; *P* < 0.01) compared to those residing in Yangon were more likely to incur catastrophic cost. Higher monthly household income over 300,000 MMKs (162 USD), was associated with a decreased risk of incurring CHE (a*OR* = 0.38; *P* < 0.01) compared to those with monthly household income less than 300,000 MMKs (162 USD). Factors such as age (*P* = 0.37), TB treatment regimen (*P* = 0.22), employment status (*P* = 0.67), comorbidities (*P* = 0.87), and sources for TB care (*P* = 0.95) were not significant in the multivariate logistic regression model (Table 5).

**Table 5:** Factors associated with catastrophic health expenditure among tuberculosis (TB) patients (*N* = 695)

	Number (%) who incurred catastrophic costs	Unadjusted regression		Adjusted regression	
Variable	Yes	*OR*	*P*-value	a*OR*	*P*-value
Age of patient in years	–	1.00 (1–1.02)	0.30	1.01 (1–1.02)	0.37
Region					
Yangon	164 (68.3)	1 [Reference]		1[Reference]	
Non-Yangon	76 (31.7)	1.64 (1.16–2.34)	**0.01**	2.58 (1.62–4.12)	**0.00**
Gender					
Male	137 (57.1)	1 [Reference]		1[Reference]	
Female	103 (42.9)	1.08 (0.79–1.49)	0.60	0.84 (0.54–1.29)	0.41
Total monthly HH income category					
≤ 300,000 MMK or 162 USD	169 (70.4)	1 [Reference]		1[Reference]	
> 300,000 MMK or 162 USD	71 (29.6)	0.33 (0.24–0.46)	**0.00**	0.38 (0.25–0.57)	**0.00**
Treatment regimen					
Initial regimen	207 (86.3)	1 [Reference]		1[Reference]	
Retreatment regimen	33 (13.8)	1.01 (0.64–1.59)	0.96	0.69 (0.38–1.25)	0.22
Employment status					
Employed with earnings	95 (39.6)	1 [Reference]		1[Reference]	
Unemployed or no Job	98 (40.8)	1.62 (1.14–2.30)	**0.01**	1.10 (0.7–1.73)	0.67
Dependent and unable to work	47 (19.6)	1.42 (0.92–2.18)	0.12	1.29 (0.71–2.35)	0.39
History of hospitalization					
Yes	34 (14.2)	12.35 (5.11–16.75)	**0.00**	14.84 (5.46–40.37)	**0.00**
No	206 (85.8)	1 [Reference]		1[Reference]	
Using coping strategy					
Yes	140 (58.3)	11.34 (7.68–15.17)	**0.00**	12.53 (8.04–19.53)	**0.00**
No	100 (41.7)	1 [Reference]		1[Reference]	
Having comorbidities					
TB only	159 (66.3)	0.77 (0.52–1.15)	0.20	1.08 (0.64–1.83)	0.77
TB and HIV, TB and DM/HTN	51 (21.3)	1 [Reference]		1[Reference]	
TB with unknown comorbidities status	30 (12.5)	0.90 (0.51–1.60)	0.73	0.94 (0.45–1.97)	0.87
Health care source					
SQH	222 (92.5)	1 [Reference]		1[Reference]	
Non-SQH	18 (7.5)	1.68 (0.87–3.21)	0.12	1.03 (0.422.55)	0.95

*DM* Diabetes mellitus; *HTN* Hypertension; *SQH* Sun quality health; *P*-value < 0.05 are described in bold

## Discussion

Despite free TB care was offered in private health care setting in Myanmar, TB patients still incurred significant cost for diagnostic, treatment and catastrophic health expenditure. In this study, CHE for TB care was 34.5% after reimbursement, which was lower than the reported rates of 52.8% [25], 65.0% [26], and 78.1% [27] in China, Nigeria, and Benin, respectively, using the same CHE measurement with different cut-offs. Both studies from China and Nigeria utilized household direct cost and income data to determine catastrophic costs, establishing a threshold of more than 40% of a household's capacity to pay or 10% of total household income. In contrast, the Benin study used conventional threshold of 10% of annual household income is used to define catastrophic health expenditure. A sensitivity analysis was carried out using a range of thresholds from 5% to 25% to understand the impact of different threshold levels on the measurement of catastrophic health expenditure. Moreover, the CHE rate of 34.5% in this study was lower than the global pooled average of 48%, which was derived from 27 countries with published survey data [3], and it was also lower than the pooled average of 135 low- and middle-income countries with meta-regression estimates 54.9% (47.0–63.2%) [28].

To our knowledge, this was the first survey on the costs incurred by TB patients in Myanmar's private sector, utilizing the tools developed by WHO for assessing the costs at household level [15, 20, 21]. The results revealed the significant CHE experienced by TB patients in the private sector in Myanmar, which is vital for achieving the end TB strategies. Additionally, it highlighted gaps in the implementation of TB policies that required improvement. In 2015 study, that primarily focused on public sector TB patients in Myanmar, CHE was defined if the proportion of total costs exceeded 20% of the annual household income. It was found that 60% of TB-affected households experienced catastrophic costs [13], with an average total cost of 759 USD, with patient time (365 USD), food costs (200 USD), and medical expenses (130 USD). The study identified lowest and second lowest household wealth quintiles and households with patients undergoing MDR-TB treatment as significant predictors of catastrophic costs [13]. In contrast, this 2022 study in Myanmar focused on private TB patients in specific regions and used different criteria for defining catastrophic costs. The findings suggested that WHO’s target of eliminating the incidence of catastrophic costs required innovations in social-protection programs. A combination of strategies will be required to reduce the costs patients incur in the trajectory between the pre-treatment phase and the end of treatment.

In this study, most costs occurred between the onset of the first symptom and treatment initiation. Similar patterns were observed in studies from Indonesia [28] and India [29], where pre-TB treatment costs were higher, possibly due to limited awareness about TB symptoms and unaware of diagnostic facilities, which prevent people from accessing multiple practitioners for diagnosis. The diagnostic costs varied globally, 3.50 USD in India [30] to 220 USD in China [31] and reported that active case finding could reduce patient costs for diagnosis.

The median total cost for the entire TB treatment phase was 53.4 USD, comprising 11.9 USD for direct medical costs and 11.6 USD for direct non-medical costs. This contrasted with other countries, where the costs were significantly higher, such as 397 USD in Cambodia [32], 758 USD in Vietnam, and 742 USD in the Dominican Republic [31]. However, in Ethiopia, the cost was lower at 115 USD [33], and in Indonesia, the median cost was 133 USD [31]. These variations highlight the diverse economic impacts of TB treatment in different settings, and these only included or mostly comprised TB patients from public health facilities [34].

The phenomena of having financial burden of OOP payments and incurred catastrophic costs were common in Myanmar. Reasons for out-of-pocket costs for TB care even after provision of free TB medicine, were due to additional nutrition intake, symptom relief medications, underlying conditions, and the need for physical rest. Many associated health-care costs were not covered in the free TB policy, for example, extra-diagnostic tests, payment for medicine for other co-existing diseases. The transportation fee was supported for the first 2 months but not for the whole phase of treatment and every visit. Besides, financial support or sickness allowance for patients was reported to be received by some patients (around 1.6–16.2 USD). Also, increased adherence led to more frequent travel and job absenteeism, which could further increase treatment costs [35].

In terms of financial burden, 34.5% of households experienced catastrophic costs due to TB care, with higher risks for poorer households and those requiring hospitalization. The percentage of non-poor households pushed into poverty due to TB-related healthcare expenses was also significant [20]. This could result in families experiencing stress, losing their savings, and having debt. To reduce the economic burden, it would be beneficial to provide transportation support, drugs for symptom relief, nutritional supplements, and compensating for lost income [36, 37]. These findings underscored the need to address broader socioeconomic consequences experienced by patients and their families [38].

The study found that unemployment was more likely to have incurred costs emphasizing the need to consider these factors in strategies for improved TB service delivery. Family members also experienced work absenteeism due to caregiving demands. This might happen because more than 50% being unemployed and dependent on the households. This supported previous studies suggesting coping costs as a proxy for catastrophic costs, underlining the importance of financial protection through insurance schemes or support during treatment [36, 37].

The findings suggested that catastrophic costs of TB were evident among those who used coping strategies. The provision of additional financial protection through insurance schemes or financial support during treatment period were needed [38] because patients did not have any form of health security coverage. In addition to the free TB medicine and other support schemes, there was a need for innovative financial protections in collaboration with various sectors, including the private sector and civil societies in Myanmar.

This study had some limitations. First, the study did not include some patients who did not receive anti-TB treatment or discontinued treatment prematurely. If this situation was caused by financial barriers, it may have the possibility of underestimating the actual level of catastrophic costs. Moreover, TB patients’ criteria such as serious illness, and various contact issues were more prevalent in economically disadvantaged households. Excluding these households may underestimate the cost burden experienced by many TB-afflicted households. Those criteria presented significant challenges in data collection for follow-up and could adversely affect response rates. Additionally, recall bias was a concern for completed treatment, as participants might have struggled to accurately remember details about their treatment or health status as well as associated costs, especially after significant time had passed or completed treatment. This may lead to systematic errors in the data, complicating our analysis. Moreover, the study population was adult TB patients, and this might overlook the significant challenges faced by families with children suffering from TB. The illness of children not only affected their health but also imposed emotional and financial burdens on parents and caregivers, who may need to take time off work for clinic visits and care.

The data collected from patients with TB were self-reported, which may be subject to recall bias. The self-reported direct non-medical expenses, household income, and food expenditure due to longer recall time were also one of the limitations. Indirect costs for patients who were not working, such as students, housewives, those who worked at home, and those who were unemployed, may have been underestimated, which might have resulted in low income and reduced capacity to pay for health care and to have experienced catastrophic costs. This finding was believed to be generalizable where similar tuberculosis burden and socio-economic structures existed. Loss of productivity could be assessed in different ways; the greatest variation arose from the different methods used to place a value on productive time lost. One methodological issue was that using a cross-sectional household survey did not capture the whole range of high and low expenditures throughout the year, therefore it was likely to note mostly short-term shocks. The cut-off for healthcare spending, calculated as a percentage of overall consumption or non-food expenditure (capacity to pay), was controversial. In this study, healthcare payments exceeding 40% of consumption expenses are categorized as catastrophic [20].

While recognizing the inherent limitations of the study, these constraints did not significantly diminish the significance of the results. The study was unique in Myanmar in reporting TB expenditure estimates in detail, comprising of costs incurred in different phases such as pre-TB diagnostic, TB diagnostic and treatment along with estimating indirect costs (loss of productivity). In addition, this study used a standardized questionnaire specifically developed for estimating patients’ costs for tuberculosis care comprehensively covering all aspects and phases for expenditure. This was among the few studies in Myanmar conducted on cost estimation for treatment of TB patient, contributing significantly to our understanding of estimating CHE among tuberculosis patients seeking care at private sector, providing valuable insights that remain relevant despite the acknowledged limitations. The study served as a valuable contribution to the existing body of knowledge.

As for future research, we recommend including a more diverse geography and population to better understand the financial impact of TB. While telephone-based data collection produced good quality data, in-person data collection at TB patients’ households could minimize the contact issues and improve the response rates experienced by the current study.

## Conclusions

Despite the country's extensive network of free TB diagnostic and treatment services, private sector TB patients, faced significant out-of-pocket expenses and catastrophic health expenditure in Myanmar. Pre-treatment cost was noted as the largest proportion compared to post treatment cost. Beyond providing free TB care, new strategies or policies are required to offset nonmedical and medical costs and ensure TB care is affordable for all TB patients.

## Data Availability

The dataset contained programmatic and sensitive clinical client information and thus, are not publicly available. Data can be made available from the authors upon reasonable request and with permission from the relevant organizations listed above.

## Abbreviations

AHRN: Asian Harm Reduction Network
a*OR*: Adjusted odd ratio
CHE: Catastrophic health expenditure
DM: Diabetes mellitus
DS-TB: Drug-susceptible TB
DR-TB: Drug-resistant TB
GP: General practitioners
Jhpiego: Johns Hopkins Program for International Education in Gynecology and Obstetrics
MAM: Medical Action Myanmar
MATA: Myanmar Anti-TB association
MDR-TB: Multi-drug-resistant TB
MMK: Myanmar Kyats
NGO: Non-governmental organization
NTP: National TB Program
Oopctp: Out-of-pocket health payments share of household capacity to pay
PPM: Public-private Mix
PPS: Probability proportional to the size
PSI: Population Services International
SQH: Sun Quality Health
SES: Social economic status
TB: Tuberculosis
USAID: United States Agency for International Development
USD: US dollar
WHO: World Health Organization
